# Impact of Public Health and Social Measures on the COVID-19 Pandemic in the United States and Other Countries: Descriptive Analysis

**DOI:** 10.2196/27917

**Published:** 2021-06-02

**Authors:** Sophia Alison Zweig, Alexander John Zapf, Hanmeng Xu, Qingfeng Li, Smisha Agarwal, Alain Bernard Labrique, David H Peters

**Affiliations:** 1 Department of Epidemiology Johns Hopkins University Bloomberg School of Public Health Baltimore, MD United States; 2 Department of International Health Johns Hopkins University Bloomberg School of Public Health Baltimore, MD United States

**Keywords:** surveillance, COVID-19, public health, health policy, global health, policy, epidemiology, descriptive epidemiology

## Abstract

**Background:**

The United States of America has the highest global number of COVID-19 cases and deaths, which may be due in part to delays and inconsistencies in implementing public health and social measures (PHSMs).

**Objective:**

In this descriptive analysis, we analyzed the epidemiological evidence for the impact of PHSMs on COVID-19 transmission in the United States and compared these data to those for 10 other countries of varying income levels, population sizes, and geographies.

**Methods:**

We compared PHSM implementation timing and stringency against COVID-19 daily case counts in the United States and against those in Canada, China, Ethiopia, Japan, Kazakhstan, New Zealand, Singapore, South Korea, Vietnam, and Zimbabwe from January 1 to November 25, 2020. We descriptively analyzed the impact of border closures, contact tracing, household confinement, mandated face masks, quarantine and isolation, school closures, limited gatherings, and states of emergency on COVID-19 case counts. We also compared the relationship between global socioeconomic indicators and national pandemic trajectories across the 11 countries. PHSMs and case count data were derived from various surveillance systems, including the Health Intervention Tracking for COVID-19 database, the World Health Organization PHSM database, and the European Centre for Disease Prevention and Control.

**Results:**

Implementing a specific package of 4 PHSMs (quarantine and isolation, school closures, household confinement, and the limiting of social gatherings) early and stringently was observed to coincide with lower case counts and transmission durations in Vietnam, Zimbabwe, New Zealand, South Korea, Ethiopia, and Kazakhstan. In contrast, the United States implemented few PHSMs stringently or early and did not use this successful package. Across the 11 countries, national income positively correlated (*r*=0.624) with cumulative COVID-19 incidence.

**Conclusions:**

Our findings suggest that early implementation, consistent execution, adequate duration, and high adherence to PHSMs represent key factors of reducing the spread of COVID-19. Although national income may be related to COVID-19 progression, a country’s wealth appears to be less important in controlling the pandemic and more important in taking rapid, centralized, and consistent public health action.

## Introduction

By the end of the first year of the pandemic, the United States had the highest global, cumulative COVID-19 case and death counts [1]. Additional challenges, such as newly emergent lineages of SARS-CoV-2 with increased transmission potential and issues in COVID-19 vaccine distribution and administration, have increased the burden on public health systems worldwide [2]. The implementation of public health and social measures (PHSMs) such as stay-at-home orders, limited gatherings, and the closure of nonessential workplaces is a crucial method for preventing and mitigating the spread of COVID-19. In spring of 2020, the Health Intervention Tracking for COVID-19 (HIT-COVID) database was developed to catalogue the global implementation and relaxation of COVID-19–related PHSMs [3]. Many other PHSM surveillance systems also collect data that can be used to inform pandemic responses [4-6]. These PHSMs have been shown to be effective, both individually and in combination, against COVID-19 globally. For example, a recent study found that from January to May 2020, limiting gatherings, closing businesses, closing schools and universities, and implementing stay-at-home orders were individually effective at reducing the time-varying reproduction number (R_t_) of SARS-CoV-2 [7]. An earlier study found that in combination, limited gathering sizes, business closures, educational institution closures, and stay-at-home orders reduced COVID-19 transmission from January to May 2020 in 11 European countries [8]. The implementation of PHSMs is a key marker of how public health systems address the COVID-19 pandemic. Poor COVID-19 outcomes in the United States of America have been attributed to a failure to consistently, quickly, and effectively implement PHSMs [9]. We sought to analyze the epidemiological evidence for the impact of PHSMs on COVID-19 transmission in the United States and compare these data to those for 10 other countries—places that provide sources of learning to the United States.

## Methods

We analyzed the timing and stringency of PHSMs that were implemented from January 1 to November 25, 2020, compared them against time series for daily case counts of COVID-19, and compared the United States to Canada, China, Ethiopia, Japan, Kazakhstan, New Zealand, Singapore, South Korea, Vietnam, and Zimbabwe.

These 10 countries were chosen for comparison with the United States based on their varying income levels and geographies and were selected as comparators for the US COVID-19 response, which was reported in a previously published, high-profile commentary on global PHSM effectiveness [9]. Canada was chosen for its comparatively lower death rate; China was chosen for being the first country affected by COVID-19 and having a large population; Japan was chosen for its older population; New Zealand was chosen for being geographically isolated; Singapore and South Korea were chosen for their geographic proximity to China; Vietnam was chosen for being a lower-middle–income country; and Ethiopia, Kazakhstan, and Zimbabwe were chosen for having less medical infrastructure and manufacturing capacity.

For ease of analysis, we focused on the following eight categories of PHSMs: border closures, contact tracing, household confinement, mandated face masks, quarantine and isolation, school closures, limited gatherings, and a state of emergency. These categories of PHSMs were chosen based on a brief literature review, data availability in the HIT-COVID database, and an exploratory data analysis. The stringency of PHSM implementation was classified in the HIT-COVID surveillance database as strongly implemented, partially implemented, or not implemented based on the specific details of each PHSM. After being abstracted from government or news websites, data on PHSM timing and stringency in the HIT-COVID database undergo internal auditing by both the person who entered the data and the database management team [3]. PHSM and case count data were derived from the HIT-COVID database, the World Health Organization PHSM global database, and the European Centre for Disease Prevention and Control [5,10]. For each country, the date and stringency of PHSM implementation were plotted against the number of daily national COVID-19 cases from January 1 to November 25, 2020. Additionally, we compared the 11 countries’ cumulative incidence rates to 40 variables that described national measures of social, demographic, economic, and health system characteristics [11-14]. The analysis was performed by using the HIT-COVID R package version 4.0.3 [15]. All data used in this analysis were publicly available, and this study did not constitute human subjects research; therefore, ethical review was not required.

## Results

According to the HIT-COVID database, from January 1 to November 25, 2020, 11,999 PHSMs were implemented in 148 countries. Of these PHSMs, 5,695 fell into the eight categories that were defined for analysis in this study. School closure was the most common measure (1592/5695, 27.95%), followed by border closures (1481/5695, 25.98%), quarantine and isolation (705/5695, 12.39%), limited gatherings (629/5695, 11.06%), household confinement (573/5695, 10.05%), face mask mandates (272/5695, 4.78%), a state of emergency (269/5695, 4.73%), and contact tracing (174/5695, 3.04%).

**Figure 1:**
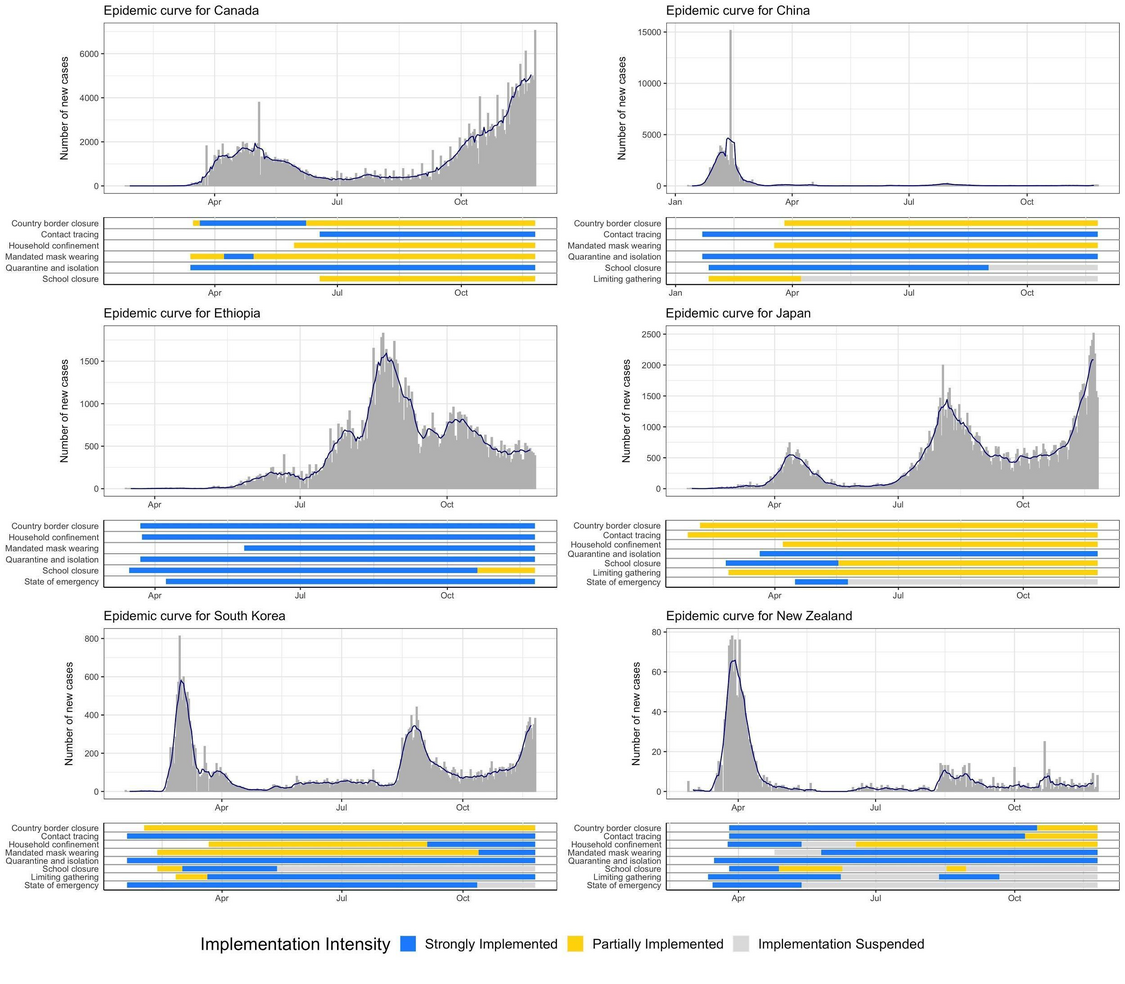
COVID-19 epidemic curves and public health and social measure timelines for China, Canada, Ethiopia, Japan, New Zealand, and South Korea (January 1 to November 25, 2020).

**Figure 2:**
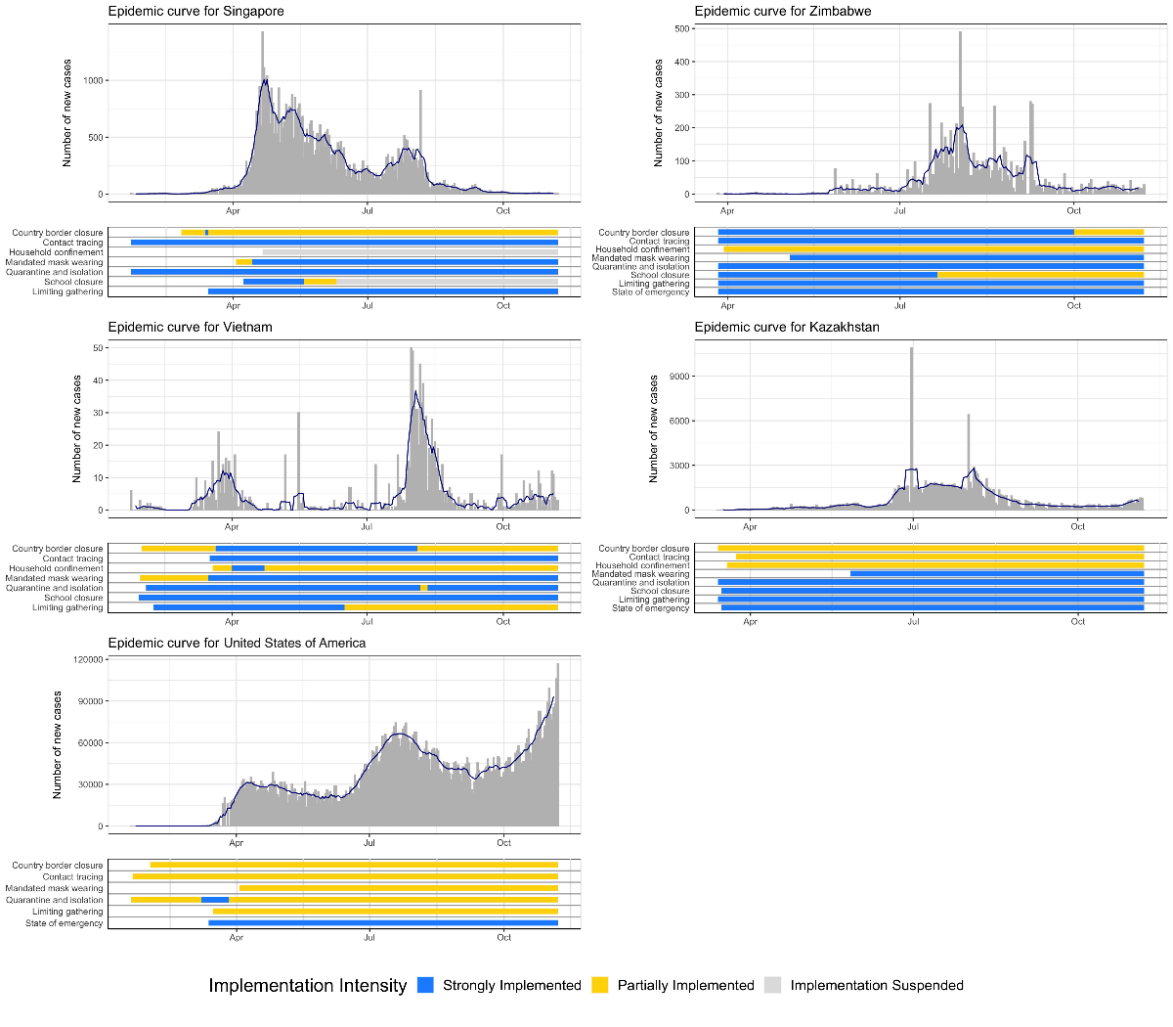
COVID-19 epidemic curves and public health and social measure timelines for Kazakhstan, Singapore, the United States, Vietnam, and Zimbabwe (January 1 to November 25, 2020).

We compared the cross-country timing and stringency of PHSMs to COVID-19 epidemic curves, as shown in Figures 1 and 2. The results show that implementing a specific package of PHSMs—quarantine and isolation, school closures, household confinement, and limited social gatherings—earlier and more stringently coincided with limited case counts and transmission durations in Ethiopia, Kazakhstan, New Zealand, South Korea, Vietnam, and Zimbabwe. Further, these countries’ case counts and transmission durations were lower than those of the United States. Singapore and South Korea implemented similar PHSMs, including less stringent household confinement, but both countries substantially lowered the epidemic curve further than the United States. China implemented fewer PHSMs for shorter durations via more targeted subnational implementation. Japan implemented strong quarantine and isolation measures along with early school closures but did not maintain the same level of stringency as that of the package of 4 PHSMs. Canada initially implemented strict PHSMs but did not maintain this level of stringency across the country. Canada is now facing a resurgent epidemic and has a relatively high cumulative COVID-19 incidence rate, though it is still much lower than that of the United States.

In contrast, the United States did not implement the package of 4 PHSMs that were shown to be effective in the 6 countries mentioned above. The United States implemented few PHSMs fully or early during the pandemic. Although the US government implemented some PHSMs early in the pandemic, these PHSMs were only partially implemented; they were not strongly implemented like the PHSMs of most of the other countries in this analysis.

Border closures, state-of-emergency declarations, and mandated mask wearing did not show strong temporal overlap with epidemic growth across the 11 countries, though some of these PHSMs may have been important in individual countries (eg, border closures in New Zealand). The adherence to mask wearing seemed to vary widely across countries.

**Figure 3:**
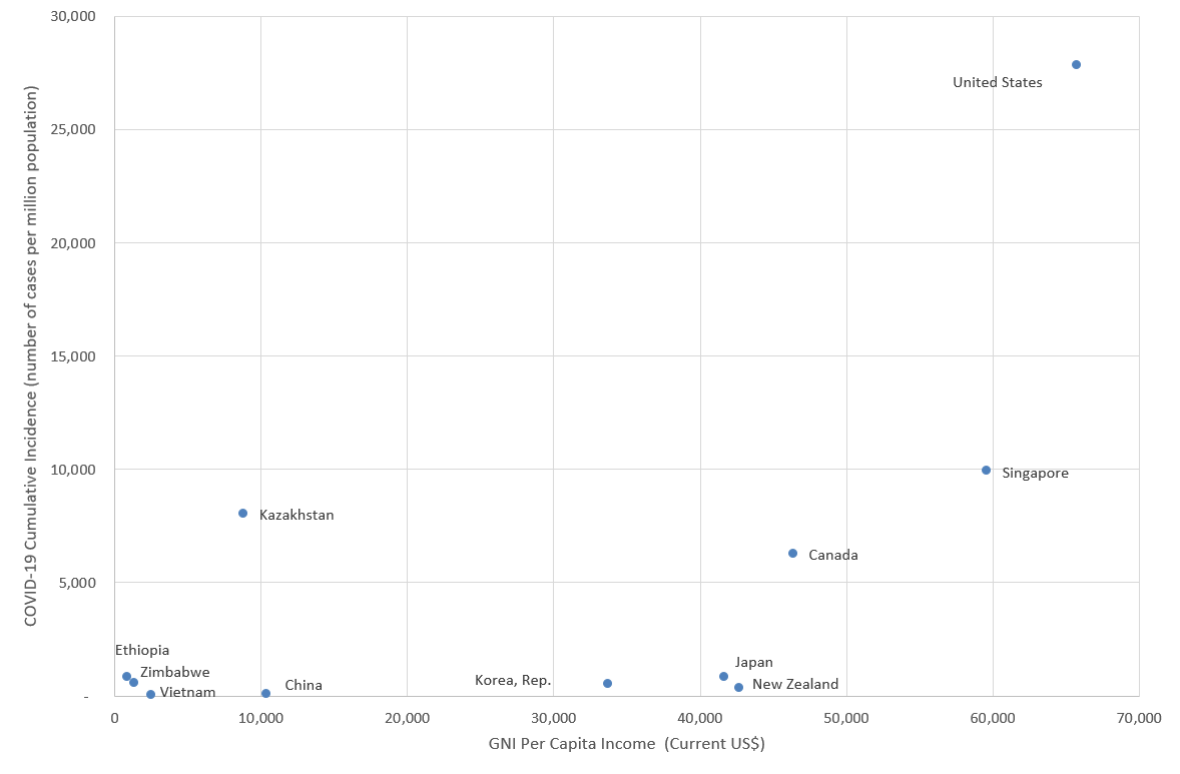
Cumulative COVID-19 incidence rates (January 1 to November 25, 2020) and national income levels. GNI: gross national income.

The 11 countries differed significantly in terms of many demographic, economic, social, and health system characteristics. These differences may contribute to their varying success. After comparing the countries’ cumulative COVID-19 incidence rates with national measures of social, demographic, economic, and health systems factors, we found only 1 positive correlation (*r*=0.624; *r*^2^=0.389) between national income (measured as gross national income per capita in US dollars) and cumulative COVID-19 incidence (Figure 3). Lower-income countries performed better than higher-income countries; the three countries with the lowest incomes had the lowest incidence rates, and the two countries with the highest incomes had the highest incidence rates.

## Discussion

### Principal Findings

Efforts to control the COVID-19 pandemic have ranged from early, strict border closures and nationwide lockdowns to less stringent individual behavior change campaigns and public health recommendations. The exact mixture of PHSM implementation approaches, timings, and intensities has varied across regions and countries and often within countries depending on their size and governance structure. Public health experts have stated that the early and stringent implementation of mitigation strategies is key to pandemic control and results in greater reductions in the number of downstream cases and fatalities [9]. One study found that globally, the timing of PHSM implementation was significantly associated with a reduction in COVID-19 transmission rates, as measured by the R_t_ ratio [16]. Our analysis suggests that earlier PHSM implementation times as well as more stringent implementation may be related to lower caseloads and transmission durations across 11 countries. The package of 4 PHSMs that temporally coincided with decreased COVID-19 case counts and transmission durations (quarantine and isolation orders, school closures, household confinement, and limits on social gatherings) has been shown to be effective in other studies, thereby providing further evidence that these specific measures help to reduce the spread of the pandemic [7,8]. An advantage of our analysis is that the HIT-COVID data set includes a measure of implementation stringency for each individual PHSM (strongly, partially, or not implemented). Most other databases classify PHSM stringency according to the strictness of behavior-related PHSMs; thus, stringency is dependent on the type of PHSM as well as PHSM implementation [6].

Implementing effective COVID-19 PHSMs requires coherent national leadership and coordination as well as necessary resources. The US government’s pandemic management has been highly fragmented. Although a more unified response has been called for by US public health leaders, the United States has a strong history of state-level policy making, which creates logistical and cultural challenges to implementing stringent national PHSMs. Additionally, cultural distrust in public health and medicine may have contributed to implementing PHSMs later than what is recommended by public health experts. Finally, while the United States is a high-income country, low hospital workforce capacity, the lack of affordable health care, and the high prevalence of preexisting conditions may increase the population's susceptibility to contracting COVID-19 [17]. A lack of resources and intervention fatigue combined with the political pressure to reopen earlier than what public health officials have recommended may have contributed to an out-of-control pandemic that has infected over 31 million Americans and has left over 560,000 dead [1]. Our findings underscore that early, coordinated implementation; consistent enforcement; and high societal adherence to an adequate implementation duration were vital to controlling COVID-19 successfully in many countries.

Our finding of a positive relationship between national income and cumulative COVID-19 incidence raises further questions. Few studies have examined the impact of country-level income differences on COVID-19 outcomes. One study found a correlation between COVID-19 mortality and gross domestic product in 106 countries [18]. Another study found that the COVID-19 mortality rate ratio between older and middle-aged populations is higher in high-income countries than in low-income countries [19]. Finally, one analysis found that national income was positively associated with COVID-19 incidence and death rates across 210 countries; however, this paper has not been peer-reviewed [20]. Our result may be explained by the timing of PHSM implementation. According to Figure 1, lower-income countries implemented the package of 4 PHSMs early, making them more effective in mitigating COVID-19 transmission. Additionally, higher-income countries may face higher COVID-19 transmission rates due to the increased availability of international air travel. It is also possible that well-funded public health reporting systems and the availability of COVID-19 testing in higher-income countries are contributing factors. Some economists have argued that the estimates of COVID-19 morbidity and mortality rates in low- and middle-income countries (LMICs) are underestimated, as demographic simulations have suggested a much higher COVID-19 toll in LMICs, and that country-level income disparities are due to the pandemic not fully spreading through LMICs [21]. Finally, country-specific characteristics such as government structure, trust in public health and medicine, and the centralization of the pandemic response play a role in COVID-19 transmission and may be related to national income. Future research should further examine the relationship between country wealth and COVID-19 outcomes.

Our analysis has several limitations. Our sample size of only 11 countries limits the power of our analysis. We were not able to capture how inadequate policies exacerbate racial and ethnic inequities in COVID-19 outcomes. In the United States, the number of cases is 2.8 times higher among Indigenous and Latinx Americans and 2.6 times higher among Black Americans compared to that number among White Americans [22]. The age-adjusted mortality rate is 3.2 times higher among Black and Latinx Americans and 3.1 times high among Indigenous Americans compared to that rate among White Americans [23]. Higher rates of death and infection among people of color in other countries further highlight the influence of structural racism on health [24]. There is an urgent need for standardized, publicly available COVID-19 data that are disaggregated by race and ethnicity. Another limitation is that this study was a descriptive analysis, which precludes us from making conclusions about causal associations. Additionally, our population-level lens cannot account for individual-level behaviors, and we cannot differentiate the individual effects of PHSMs that were implemented concertedly in time and space. This may have contributed to the weak relationships observed between face mask policies and case counts. Finally, the PHSMs that were logged in the HIT-COVID database did not represent all PHSMs that were actually implemented during the study time period, as there may have been PHSMs that were not captured in the database.

### Conclusion

The COVID-19 pandemic has thrown societies into disorder and has challenged us to rethink what characterizes a strong health system. Although adequate resources and robust health care systems are necessary for an effective pandemic response, our analysis highlights that rapid, decisive, stringent, national, and consistent public health interventions are crucial for preventing disaster and chaos. This descriptive analysis highlights country-level differences in PHSM implementation and COVID-19 transmission. Our work supports existing studies that have reported on the association between PHSM implementation timing and stringency and COVID-19 outcomes. Furthermore, this paper raises important questions for future research on the impact of socioeconomic factors on country-level outcomes of COVID-19 transmission.

## Abbreviations

HIT-COVID: Health Intervention Tracking for COVID-19
LMIC: low- and middle-income countries
PHSM: public health and social measure
